# Inebriety: Its Etiology, Pathology, Treatment, and Jurisprudence

**Published:** 1888-06

**Authors:** 


*Inebriety: its Etiology, Pathology, Treatment, and Jurisprudence.* Pp. 415. London: H. K. LEWIS. 1888.

We commend this work to the favourable notice of our readers as a sympathetic, sensible, and scientific treatise on the whole painful subject of Inebriety. The author has evidently an extensive experience in the disease—for disease it is—and he has a special and intimate knowledge of the jurisprudence of the subject, which is treated with fulness and perspicacity. The numerous cases related are peculiarly interesting; and it is difficult to lay down the work till one has finished it. The last sentence in the preface reads: "I am also not without hope that the work may be found serviceable by my professional colleagues." We should have liked to believe that this was his chief, if not his only, motive.

